# A constitutive serine protease inhibitor suppresses herbivore performance in tea (*Camellia sinensis*)

**DOI:** 10.1093/hr/uhad178

**Published:** 2023-09-01

**Authors:** Meng Ye, Chuande Liu, Nana Li, Chenhong Yuan, Miaomiao Liu, Zhaojun Xin, Shu Lei, Xiaoling Sun

**Affiliations:** Key Laboratory of Biology, Genetics and Breeding of Special Economic Animals and Plants, Ministry of Agriculture and Rural Affairs, National Center for Tea Plant Improvement, Tea Research Institute, Chinese Academy of Agricultural Sciences, Hangzhou 310008, China; Key Laboratory of Biology, Genetics and Breeding of Special Economic Animals and Plants, Ministry of Agriculture and Rural Affairs, National Center for Tea Plant Improvement, Tea Research Institute, Chinese Academy of Agricultural Sciences, Hangzhou 310008, China; Key Laboratory of Biology, Genetics and Breeding of Special Economic Animals and Plants, Ministry of Agriculture and Rural Affairs, National Center for Tea Plant Improvement, Tea Research Institute, Chinese Academy of Agricultural Sciences, Hangzhou 310008, China; Key Laboratory of Biology, Genetics and Breeding of Special Economic Animals and Plants, Ministry of Agriculture and Rural Affairs, National Center for Tea Plant Improvement, Tea Research Institute, Chinese Academy of Agricultural Sciences, Hangzhou 310008, China; Key Laboratory of Biology, Genetics and Breeding of Special Economic Animals and Plants, Ministry of Agriculture and Rural Affairs, National Center for Tea Plant Improvement, Tea Research Institute, Chinese Academy of Agricultural Sciences, Hangzhou 310008, China; Key Laboratory of Biology, Genetics and Breeding of Special Economic Animals and Plants, Ministry of Agriculture and Rural Affairs, National Center for Tea Plant Improvement, Tea Research Institute, Chinese Academy of Agricultural Sciences, Hangzhou 310008, China; Key Laboratory of Biology, Genetics and Breeding of Special Economic Animals and Plants, Ministry of Agriculture and Rural Affairs, National Center for Tea Plant Improvement, Tea Research Institute, Chinese Academy of Agricultural Sciences, Hangzhou 310008, China; Key Laboratory of Biology, Genetics and Breeding of Special Economic Animals and Plants, Ministry of Agriculture and Rural Affairs, National Center for Tea Plant Improvement, Tea Research Institute, Chinese Academy of Agricultural Sciences, Hangzhou 310008, China

## Abstract

Protease inhibitors promote herbivore resistance in diverse plant species. Although many inducible protease inhibitors have been identified, there are limited reports available on the biological relevance and molecular basis of constitutive protease inhibitors in herbivore resistance. Here, we identified a serine protease inhibitor, *CsSERPIN1*, from the tea plant (*Camellia sinensis*). Expression of *CsSERPIN1* was not strongly affected by the assessed biotic and abiotic stresses. *In vitro* and *in vivo* experiments showed that CsSERPIN1 strongly inhibited the activities of digestive protease activities of trypsin and chymotrypsin. Transient or heterologous expression of *CsSERPIN1* significantly reduced herbivory by two destructive herbivores, the tea geometrid and fall armyworm, in tea and *Arabidopsis* plants, respectively. The expression of *CsSERPIN1* in *Arabidopsis* did not negatively influence the growth of the plants under the measured parameters. Our findings suggest that *CsSERPIN1* can inactivate gut digestive proteases and suppress the growth and development of herbivores, making it a promising candidate for pest prevention in agriculture.

## Introduction

Insect herbivores of tea plants are estimated to cause an 11%–55% loss in yield, which is worth US$500 million to US$1 billion worldwide [1]. Genetically modifying plants to improve their resistance to insect pests is an environment-friendly strategy for pest control. One possible approach is the use of defense-related metabolites, such as protease inhibitors [2, 45]. Plant protease inhibitors can inhibit digestive proteases of insect herbivores and, consequently, reduce the uptake of necessary amino acids for herbivore growth and development [2, 3].

Serine protease inhibitor (SERPIN) is one of the largest superfamilies of protease inhibitors in plants [4]. Most SERPINs inhibit serine proteases, but some also inhibit cysteine proteases [5]. In addition to their involvement in physiological processes, SERPINs play critical roles in defense against herbivores by suppressing the enzymatic activity in the insect alimentary canal, which inhibits the absorption of vegetable proteins [6]. In past decades, a number of transgenic plants overexpressing *SERPINs* have been generated to test the increase in plant resistance against insect herbivores. For instance, AtSerpin1 inhibits cysteine and serine protease activities of several leaf herbivores in Arabidopsis (*Arabidopsis thaliana*). Transgenic *Arabidopsis* lines overexpressing *AtSerpin1* dramatically reduce the larval growth of cotton leafworm (*Spodoptera littoralis*) [7]. Expression of a barley *SERPIN* in tomato shows increased resistance to tomato leaf miner (*Tuta absoluta*) [8]. Heterologous expression of two fused protease inhibitors, a maize *SERPIN* and a potato carboxypeptidase inhibitor, in rice enhances defense against the striped stem borer (*Chilo suppressalis*) [9]. While plenty of *SERPINs* obtained from crop plants have been demonstrated to confer resistance to a wide variety of herbivores [7, 9–12], it remains unclear whether and how *SERPINs* influence herbivore resistance in tea plants.

Generally, inducible protease inhibitors function as part of plant active defense responses, as they are elicited upon herbivory-related signal perception [6]. They are expressed and accumulated in specific tissues that are being or going to be attacked [8, 13]. This tight spatial–temporal regulation not only allows plants to respond specifically and efficiently, but also controls plant defensive investment in a cost-saving way [14, 46]. However, this transcriptional and posttranscriptional control of inducible protease inhibitors is more susceptible to environmental factors, and it can even provide an opportunity for adapted herbivores to avoid effective defenses, leading to an unstable resistance phenotype. For instance, the spider mite (*Tetranychus evansi*) manipulates the expression and activity of inducible protease inhibitors, thereby suppressing the defense responses of tomato plants [15]. Thus, we argue that constitutively expressed protease inhibitors may be crucial or even more reliable in guaranteeing effective defenses against herbivores. Although many inducible protease inhibitors have been identified, there are limited reports available on the biological relevance and molecular basis of constitutive protease inhibitors in herbivore resistance.

**Figure 1:**
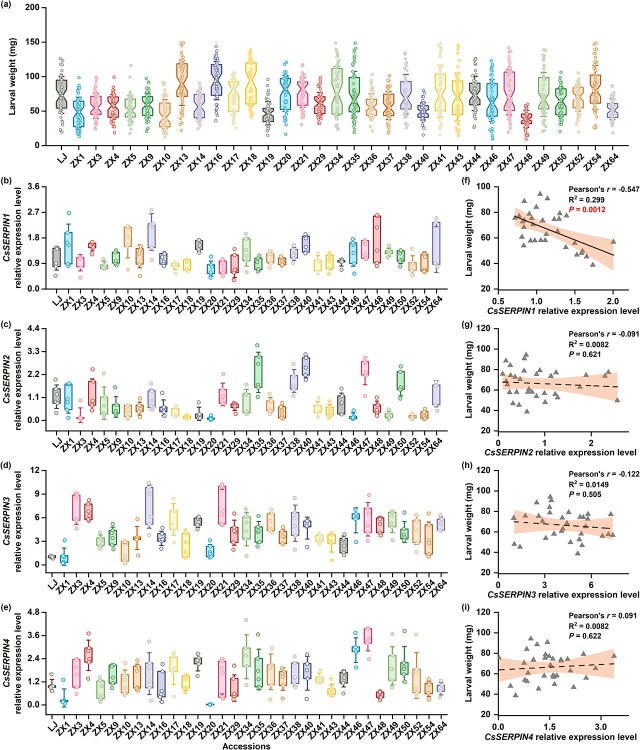
Expression of *CsSERPIN1* correlates with herbivore growth in tea plants. (**a**) Larval weight gain of tea geometrid feeding on different tea accessions for 7 days (± SD, *n* = 51–56). (**b**–**e**) *CsSERPIN1*, *CsSERPIN2*, *CsSERPIN3*, and *CsSERPIN4* expression levels in different tea accessions (± SD, *n* = 6). For the detailed statistical information, refer to Tables S1 andS2 (see online supplementary material). (**f**–**i**) Correlations between the expression of *CsSERPIN1*, *CsSERPIN2*, *CsSERPIN3*, and *CsSERPIN4* genes, and larval weight gain. The Pearson’s product–moment *r*, R^2^, and *P* values of correlations are indicated.

Here, we identified a novel constitutively expressed serine protease inhibitor, *CsSERPIN1* from tea plants. We characterized its expression patterns under both abiotic and biotic stresses, and determined its inhibitory activities both *in vitro* and *in vivo*. We clarified how CsSERPIN1 protects plants against two destructive pests, tea geometrid (*Ectropis obliqua*) and fall armyworm (*Spodoptera frugiperda*) by using a reverse genetic approach. Our results suggest that *CsSERPIN1* has great potential for exploitation in pest control in agriculture.

## Results

### *CsSERPIN1* may be involved in tea plant defenses against herbivores

To determine the potential roles of *SERPINs* in herbivore resistance, we first quantified the larval growth of a destructive pest, tea geometrid, on 33 tea accessions. We observed variation in herbivore performance among different tea accessions (Fig. 1a). We then searched the annotated *SERPIN* genes in the tea genome, and identified only four genes with an intact serpin domain (PF00079). These four *SERPINs* belong to the MEROPS inhibitor family I4, clan ID. Their serpin domain consists of nine α-helices and three β-sheets, along with a semi-conserved reactive center loop that contains the active site recognized by the target protease [6]. The full-length sequences of the four identified *SERPINs* (referred to as *CsSERPIN1*, *CsSERPIN2*, *CsSERPIN3*, and *CsSERPIN4*) were cloned and validated by reverse transcription PCR and Sanger sequencing. Our phylogenetic analysis of these four *SERPINs*, along with 24 herbivore-related *SERPINs* from 14 plant species, revealed that *CsSERPIN1* and *CsSERPIN2* were homologous to *AtSerpin1* in Arabidopsis (Fig. S1, see online supplementary material). We further quantified the transcriptional levels of these four *SERPIN* genes in the second and third fully expanded leaves of 33 tea accessions. These specific leaf positions were chosen as they were utilized for the above herbivore resistance assessment. We found that the expression level of *CsSERPIN1*, but not the remaining three *SERPIN* genes, was negatively correlated with herbivore growth (Fig. 1b–i; Tables S1 andS2, see online supplementary material). These data suggest that the expression of *CsSERPIN1* is associated with the resistance to tea geometrid.

### *CsSERPIN1* is a constitutively expressed serine protease inhibitor

**Figure 2:**
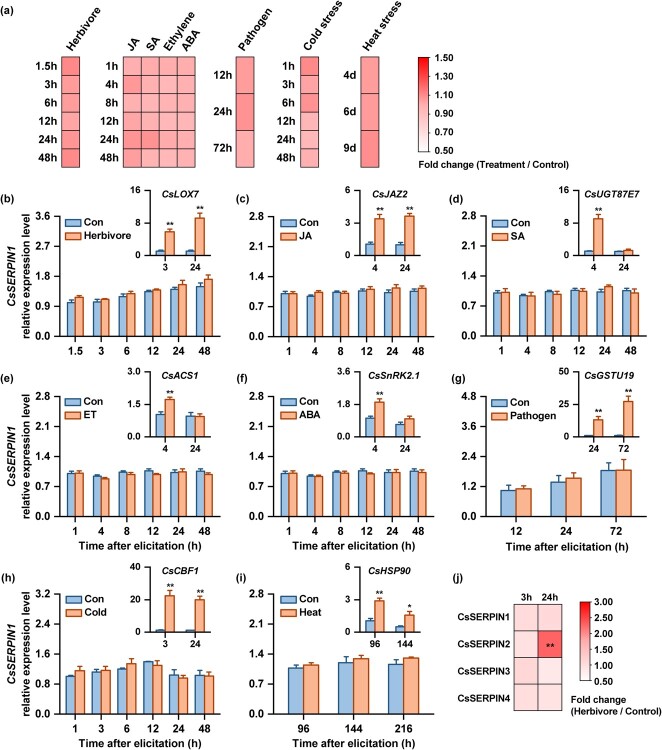
Expression pattern of *CsSERPIN1* under different treatments. (**a**) Heatmaps of *CsSERPIN1* expression under herbivore attack, jasmonic acid (JA), salicylic acid (SA), ethylene, abscisic acid (ABA), pathogen infection, cold and heat stress treatments. The color intensity indicates the fold changes of the *CsSERPIN1* expression in treated plants relative to control plants (*n* = 3–6). (**b**–**i**) The detailed expression patterns of *CsSERPIN1* (+ SE, *n* = 3–6) under various treatments, including herbivore attack (**b**), treatment with jasmonic acid (JA, **c**), salicylic acid (SA, **d**), ethylene (**e**), abscisic acid (ABA, **f**), pathogen (**g**), cold (4°C, **h**), or heat (38°C, **i**) stress, at different time points. Inserts are included to show the expression levels of marker genes for each treatment as positive controls. Asterisks indicate significant differences between treatments at the same time points (two-way ANOVA followed by pairwise comparisons through FDR-corrected LSMeans; ^*^P < 0.05; ^**^P < 0.01). (**j**) Heatmaps of *CsSERPINs* expression under herbivore attack for 3 h and 24 h. The color intensity indicates the fold changes of *CsSERPINs* expression in treated plants relative to control plants using RNA sequencing data (*n* = 5). For detailed information, refer to Data S1 (see online supplementary material).

As many defense-related *SERPINs* are induced in vegetative tissues by insect herbivores and defense-related signaling [16–18], we first investigated whether *CsSERPIN1* expression is influenced by herbivory using QRT-PCR. *CsSERPIN1* transcript levels were not affected by herbivore attack or herbivore-related signaling molecules, including jasmonic acid (JA), salicylic acid (SA), ethylene, and abscisic acid (ABA) (Fig. 2a–f). We then challenged tea plants with a regularly encountered pathogen, *Colletotrichum gloeosporioides*, but found this pathogen infection did not influence *CsSERPIN1* expression either (Fig. 2a and g). To further test whether abiotic stresses could alter the expression of *CsSERPIN1*, we treated tea plants with cold (4°C) or heat (38°C) stresses and examined *CsSERPIN1* transcription levels. However, these abiotic stresses did not significantly change the expression of *CsSERPIN1* (Fig. 2a and h–i). To rule out the possibility that the minimally affected expression of *CsSERPIN1* was due to experimental false negatives, we measured the expression levels of eight genes: *CsLOX7* [19], *CsJAZ2* [20], *CsUGT87E7* [21], *CsACS1* [22], *CsSnRK2.1* [23], *CsCBF1* [24], *CsHSP90* [25], and *CsGSTU19* [26], which are known to exhibit a robust response to different treatments. We observed strong induction of these positive control genes under each treatment in our experiments (Fig. 2b–i).

To further consolidate the expression pattern of *CsSERPIN1*, we conducted a transcriptomic analysis of tea plants after herbivore attack using RNA sequencing. We found that *CsSERPIN1*, along with *CsSERPIN3* and *CsSERPIN4*, were not affected at 3 h and 24 h after herbivore attack, while *CsSERPIN2* was significantly induced at 24 h (Fig. 2j; Data S1, see online supplementary material). *CsSERPIN1* exhibited significantly higher expression levels compared to the other 10 detected *SERPINs*, including *CsSERPIN2*, *CsSERPIN3*, and *CsSERPIN4* (Data S1, see online supplementary material). We also observed strong induction of the positive control gene *CsLOX7* at 3 h and 24 h after herbivory, which was consistent with the results above (Data S1, see online supplementary material). Therefore, *CsSERPIN1* is abundantly expressed in tea plants, and its expression pattern under our assessed biotic and abiotic stresses is highly reliable and not a result of false-negative outcomes.

To exclude the effects of host genotype on *CsSERPIN1* expression, we quantified its transcript levels in 33 tea accessions after herbivore attack. Consistently, *CsSERPIN1* was not affected in each of the above accessions (Fig. S2, see online supplementary material). Thus, *CsSERPIN1* might be a constitutively expressed *SERPIN*, and it is possible that its transcriptional regulation is minimally affected by biotic and abiotic stresses.

### CsSERPIN1 is an active serine protease inhibitor *in vitro*

**Figure 3:**
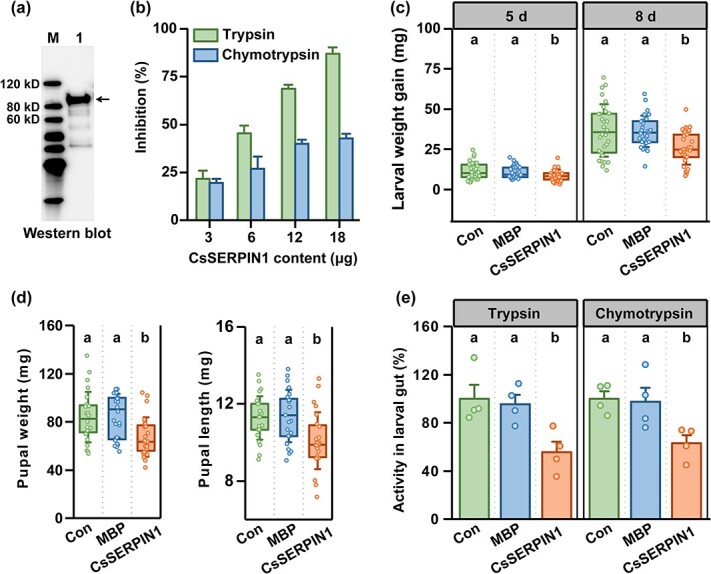
CsSERPIN1 suppresses larval performance and protease activities *in vitro* and *in vivo.* (**a**) Immunoblot analysis of purified CsSERPIN1 protein. The anti- maltose-binding protein (MBP) monoclonal antibody was used to detect the CsSERPIN1-MBP fusion protein. (**b**) The inhibitory activity of *CsSERPIN1* against trypsin and chymotrypsin enzymes *in vitro* (+ SE, *n* = 4). (**c**, **d**) Larval weight gain (**c**), pupal weight and length (**d**) of tea geometrid fed on artificial diet containing purified CsSERPIN1 or MBP, or control (Con) diet (± SD, *n* = 24–30). (**e**) Gut trypsin and chymotrypsin activities of tea geometrid fed on artificial diet containing purified CsSERPIN1 or MBP, or control diet (+ SE, *n* = 4). Statistical significance for each comparison is indicated by different letters (one-way ANOVA, *P* < 0.05).

To investigate whether *CsSERPIN1* has the inhibitory activity, we expressed it in *Escherichia coli*, and purified the protein using an affinity method (Fig. 3a). Next, we tested the inhibitory effects of purified CsSERPIN1 on the activities of two digestive proteases, chymotrypsin and trypsin, *in vitro* (Fig. 3b). Remarkably, trypsin activity was highly susceptible to CsSERPIN1, with inhibition ranging from 20–87% depending on the amount introduced. CsSERPIN1 also caused more than 20% inhibition of chymotrypsin activity. These results indicate that CsSERPIN1 is an active protease inhibitor *in vitro* and that chymotrypsin is much less susceptible than trypsin to CsSERPIN1.

### CsSERPIN1 inhibits larval performance and protease activity *in vivo*

To further evaluate the potential of CsSERPIN1 to inhibit larval digestive proteases, we introduced the purified CsSERPIN1 protein into an artificial diet. The tea geometrid larvae fed on the diets containing CsSERPIN1 gained much less weight and produced smaller pupae than those fed on control (Con) or maltose-binding protein (MBP)-containing diets (Fig. 3c and d). To investigate the underlying physiology, we dissected larvae at the end of the feeding assay and analysed the protease activity in their gut. We found the larvae fed on the diet incorporating CsSERPIN1 had much lower chymotrypsin and trypsin activities than those fed on the control diet (Fig. 3e). These findings suggest that CsSERPIN1 could inactivate larval digestive proteases *in vivo*, thereby suppressing larval performance.

### Overexpression of *CsSERPIN1* increases tea resistance to tea geometrid larvae

**Figure 4:**
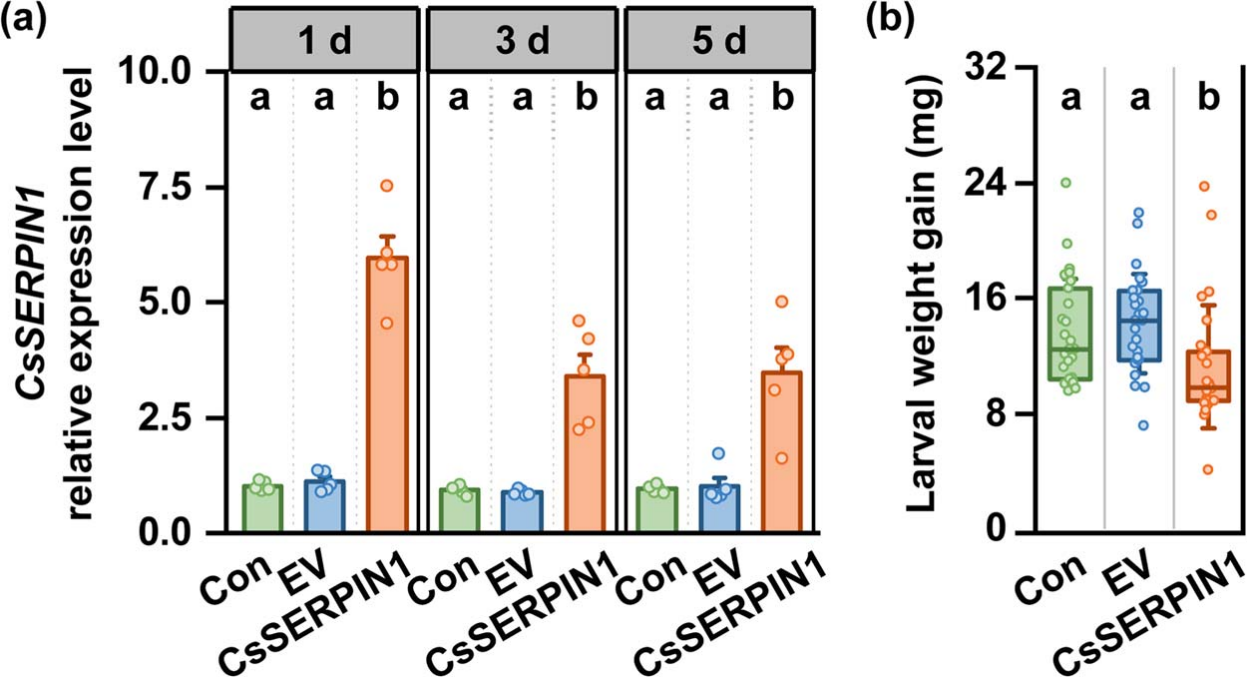
Overexpression of *CsSERPIN1* enhances tea resistance to tea geometrid. (**a**) *CsSERPIN1* expression levels in tea plant leaves with *Agrobacterium*-mediated transient overexpression (+ SE, *n* = 5). EV, empty vector. (**b**) Weight gain of tea geometrid larvae feeding on control, empty vector (EV) and *CsSERPIN1*-overexpressing tea plants (± SD, *n* = 26). Statistical significance for each comparison is indicated by different letters (one-way ANOVA, *P* < 0.05).

To investigate the role of *CsSERPIN1* in plant defense against herbivores, we transiently overexpressed *CsSERPIN1* in tea plants with *Agrobacterium tumefaciens*-mediated transformation. QRT-PCR analysis showed that the overexpression of *CsSERPIN1* persisted for at least 5 days post infiltration (Fig. 4a). Furthermore, the larvae fed on *CsSERPIN1* overexpression plants grew more slowly than larvae fed on control or empty vector (EV) transformed plants (Fig. 4b). CsSERPIN1, thus, enhances tea resistance to herbivores.

### Heterologous expression of *CsSERPIN1* increases herbivore resistance in Arabidopsis

**Figure 5:**
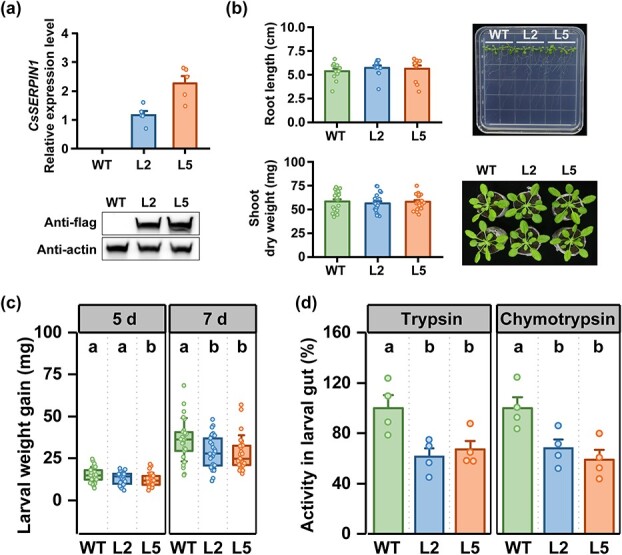
Heterologous expression of *CsSERPIN1* inhibits larval growth and gut protease activities in Arabidopsis. (**a**) Expression levels (top panel, + SE, *n* = 5) and protein accumulation (bottom panel) of CsSERPIN1 in wild-type (WT) Arabidopsis plants and *CsSERPIN1*-heteroexpressed lines (L2 and L5). The immunoblotting was performed using a flag antibody to detect CsSERPIN1, or an actin antibody to detect actin as a loading control. (**b**) Root length and shoot biomass of WT Arabidopsis and *CsSERPIN1*-heteroexpressed lines (left two panels, + SE, *n* = 12). Growth phenotypes of 12- and 30-day-old WT and *CsSERPIN1-*heteroexpressed plants are shown (right two panels). (**c**) Larval weight gain of fall armyworms fed on WT and *CsSERPIN1-*heteroexpressed plants (± SD, *n* = 25). (**d**) Gut trypsin and chymotrypsin activities of fall armyworms fed on WT and *CsSERPIN1-*heteroexpressed plants (+ SE, *n* = 4). Statistical significance for each comparison is indicated by different letters (one-way ANOVA, *P* < 0.05).

As stable transformation methods are currently unavailable for tea plants, we heteroexpressed *CsSERPIN1* in Arabidopsis to further assess its suppressive effects on herbivore growth. We verified the expression levels of *CsSERPIN1* in two homozygous lines (L2 and L5) using QRT-PCR and immunoblot assays (Fig. 5a). These two transgenic lines showed indistinguishable growth and morphology from wild-type (WT) plants, as indicated by the measured parameters (Fig. 5b). Lepidopteran larvae, fall armyworms, were allowed to fed on the *CsSERPIN1-*heteroexpressed and WT plants, and the increase in larval weight was determined. The weight gain was significantly lower on both day 5 and day 7 for fall armyworm larvae that fed on the *CsSERPIN1-*heteroexpressed lines compared to those on WT plants (Fig. 5c). Additionally, we found that larvae feeding on *CsSERPIN1-*heteroexpressed plants had much lower gut trypsin and chymotrypsin activities than those feeding on WT plants (Fig. 5d). Taken together, these results suggest that the inhibitory effects of CsSERPIN1 on herbivores extend beyond tea plants.

### CsSERPIN1 contributes to herbivore resistance variation of tea plants

**Figure 6:**
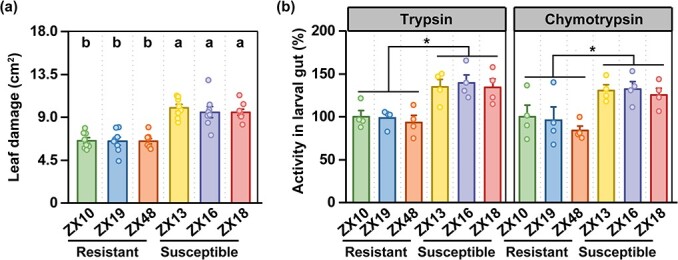
CsSERPIN1 contributes to herbivore resistance variation of tea plants. (**a**) Leaf area consumed by tea geometrid larvae feeding on three resistant (ZX10, ZX19, and ZX48) and three susceptible (ZX13, ZX16, and ZX18) tea accessions (+ SE, *n* = 8). (**b**) Gut trypsin and chymotrypsin activities in tea geometrid larvae feeding on the three resistant and three susceptible tea accessions, respectively (+ SE, *n* = 4). Statistical significance for each comparison is indicated by different letters or asterisks (one-way ANOVA, *P* < 0.05).

We hypothesized that the growth variation of tea geometrid on the 33 accessions might be due to the altered gut protease activities mediated by CsSERPIN1. To test this, a group of tea geometrid larvae were allowed to feed on the three most resistant (ZX10, ZX19, and ZX48) and three most susceptible (ZX13, ZX16, and ZX18) accessions identified above, respectively, and the activities of trypsin and chymotrypsin in larval gut were quantified. The larvae feeding on resistant accessions consumed much fewer leaves compared to those feeding on susceptible accessions (Fig. 6a), indicating that our resistance phenotype assessment was sufficiently reliable. Consistent with our hypothesis, the gut trypsin and chymotrypsin activities were much higher after feeding on susceptible accessions compared to those on resistant accessions (Fig. 6b). Together, CsSERPIN1 is implicated in the herbivore resistance variation of tea plants.

## Discussion

Inducible protease inhibitors have been shown to influence plant defenses against herbivores, but the role of constitutive protease inhibitors in herbivore resistance is still unclear. Our study helps fill these gaps by characterizing a novel constitutively expressed serine protease inhibitor, CsSERPIN1, which inactivates digestive proteases *in vitro* and *in vivo*, and consequently, suppresses the growth and development of herbivores in both *Arabidopsis* and tea plants.

While some herbivore-related protease inhibitors are induced by herbivore attack or herbivory-related signaling [16–18], our results show that the transcriptional levels *CsSERPIN1* were minimally affected by biotic and abiotic stresses. Herbivore attack, pathogen infection, cold, heat, as well as defense-related signaling molecule treatments, did not alter the expression of *CsSERPIN1*. The expression pattern of *CsCERPIN1* was confirmed by the transcriptomic analysis after herbivory using RNA sequencing. Furthermore, we found that this unaffected pattern was independent of tea genotypes, suggesting that *CsSERPIN1* might be a constitutive gene in tea plants. However, we cannot rule out the possibility that other untested factors may influence the expression of *CsSERPIN1*.

The effects of *SERPINs* on insect digestive protease activity have been extensively studied [8, 9, 16, 27]. Similarly, our *in vitro* study shows that CsSERPIN1 can inhibit trypsin and chymotrypsin proteases. Feeding assays with artificial diet and heteroexpression plants further confirmed the inhibitory ability of CsSERPIN1 in the gut of tea geometrids and fall armyworms. Given that serine proteases have been found in several insect orders including Lepidoptera, Diptera, Orthoptera, Hymenoptera, and Coleoptera [2, 28], it is quite possible that CsSERPIN1 can suppress the growth of various herbivores. However, more research is necessary to confirm this possibility. Besides serine proteases, some SERPINs could inhibit the cysteine proteases. For instance, AtSerpin1 can significantly suppress the activities of cathepsin B and L protease in aphids. Feeding assays with artificial diet indicate that AtSerpin1 is toxic to aphid nymphs with 50% mortality [7]. Thus, it would be interesting to test whether CsSERPIN1 has the similar activity and effects on herbivores that mainly rely on cysteine proteases for digestion in future studies.

In plants, serine proteolysis is a key cellular process that is carried out by a diverse group of serine proteases [29]. SERPINs are essential for the regulation of endogenous serine proteases, enabling precise control of proteolytic processes that ultimately reflect on proper plant growth and development [30]. Intriguingly, we did not observe any differences in plant growth mediated by *CsSERPIN1*. *CsSERPIN1-*heteroexpressed *Arabidopsis* plants showed similar root length and biomass to WT plants. The lack of clear phenotypic differences suggests that *CsSERPIN1* either has only marginal effects on plant growth or that its influence on plant growth is not reflected in the assessed parameters. Similarly, silencing *SERPIN SPI2a*, *SPI2b*, or *SPI2c* also does not influence the growth and development of Arabidopsis [16]. Nevertheless, more detailed experiments should be conducted to assess whether *CsSERPIN1* impacts plant growth and development.

In our study, CsSERPIN1 reduced the performance of two lepidopteran species. Feeding assays with artificial diet revealed that CsSERPIN1 interfered with the growth and pupation of tea geometrid. Transient overexpression of *CsSERPIN1* in tea plants further confirmed its suppressive effects on larval growth. Notably, we were unable to include the key experiment to prove the suppressive effects of *CsSERPIN1*, where loss of function of *CsSERPIN1* would result in increased larval weight gain, as the stable transformation technology is currently unavailable in tea plants. Instead, we attempted to transiently knock down *CsSERPIN1* with antisense oligodeoxynucleotides, but were unsuccessful, probably because the methods can vary depending on the properties of target genes. Nevertheless, further investigation of knock out/down of *CsSERPIN1* and its impact on herbivores will be necessary in future studies. Additionally, heterologous expression of *CsSERPIN1* in Arabidopsis demonstrated its notable reduction ability in the weight gain of fall armyworms. These results are consistent with previous reports that have highlighted the importance of *SERPINs* in reducing the performance of lepidopteran herbivores, either when engineered into transgenic plants or introduced into artificial diets [7, 8, 16]. While CsSERPIN1 has been observed to significantly suppress herbivores growth, it is important to note that plants have a variety of defensive compounds and multiple mechanisms to defend against herbivores. CsSERPIN1 only plays a role in one aspect of this complex defense system, rather than being the sole determinant of it. Because the resistant tea accessions could have multiple pathways to resist herbivores, including independent mechanisms involving *CsSERPIN1*, a detailed dissection of the defense mechanisms in the resistant tea accessions may offer valuable insights into the specific role of *CsSERPIN1* within the intricate defense system. This understanding could provide a valuable source of durable resistance for developing new tea cultivars.

Emerging studies show that lepidopteran herbivores can adjust their digestive proteolytic processes to counteract the inhibitory activities of plant protease inhibitors [31–33]. To overcome this herbivore adaptation, researchers suggest that it may be essential to integrate more than one inhibitor [11, 12, 34]. Therefore, in an agricultural context, while CsSERPIN1 could reduce herbivore attack, it will be wise to combine it with other protease inhibitors or protease inhibitor families, such as inducible protease inhibitors, to achieve irreversible effects on target herbivores.

Taken together, although many plant protease inhibitors have been identified and proven to play an important role in plant defense, to the best of our knowledge, *CsSERPIN1* is the first described constitutive *SERPIN* involved in herbivore resistance in tea plants. As *CsSERPIN1* is not influenced by environmental factors and does not appear to negatively affect plant growth, it has significant potential for use in herbivore control.

## Materials and methods

### Plants and insects

To investigate the involvement of *CsSERPIN1* in herbivore resistance, we used tea (*Camellia sinensis*) and Arabidopsis (*A. thaliana*) plants in this study. All 33 tea accessions were 3-year-old plants, grown at Shengzhou Experimental Station in Zhejiang Province, China (120°48′46″E, 29°44′45″N). These tea accessions, except for the ‘Longjing 43’ (LJ) accession, are our in-house breeding materials, which were carefully selected from various local tea populations across China. Some of them are still undergoing the breeding process and do not have assigned accession numbers yet. The detailed information on the three most resistant and susceptible tea accessions studied is listed in Table S3 (see online supplementary material). The 3-year-old tea cultivar ‘LJ’ was used for *CsSERPIN1* expression analysis and transient overexpression assays. Healthy tea plants of similar size were selected for experiments.

We generated *CsSERPIN1*-heteroexpressed Arabidopsis plants (L2 and L5) using an established protocol described in the section ‘Heterologous expression of *CsSERPIN1* in Arabidopsis’. Arabidopsis seeds were germinated on MS medium and grown for 10 days in MS medium before being transferred individually into round pots with commercial potting soil in a climate chamber (23°C, 50% relative humidity, 12 h/12 h photoperiod). Thirty-day-old wild-type plants and heteroexpressed lines were used for phenotype assessment and herbivore resistance assays.

Tea geometrid (*E. obliqua*) larvae were collected from natural tea plantation, and reared in a climate chamber (26°C, 70% relative humidity, 12:12 h photoperiod).

### Herbivore resistance evaluation of tea accessions

To evaluate the herbivore resistance of different tea accessions, we used larval growth and leaf consumption area as parameters. The second and third fully expanded leaves with short stems of each accession were freshly detached and placed into a hydrated floral foam for moisturizing. Leaves, together with the floral foam, were then put into a 10 cm square petri dish. Groups of seven three-day-old tea geometrid caterpillars with similar length were selected and pre-starved for 6 h before being introduced into the petri dish. The leaves were replaced once a day to ensure that caterpillars have sufficient fresh leaves. Larval weight was measured seven days after the start of assay (*n* = 51–56). To quantify the consumed leaf aera, the remaining leaf after larvae feeding were scanned, and the lost area was assessed (*n* = 8).

### *CsSERPIN1* cloning and characterization

The cDNA sequence of *CsSERPIN1* was amplified using the primers CsSERPIN1-F and CsSERPIN1-R, which were designed based on the *CsSERPIN1* sequence (NCBI accession no. LOC114291199) and are listed in Table S4 (see online supplementary material). The amplified product was cloned into a pEASY-blunt zero vector (TransGen Biotech, Beijing, China) and subsequently sequenced. Structural domain analysis was performed with SMART (Simple Modular Architecture Research Tool, http://smart.embl-heidelberg.de) and PFAM (http://pfam.xfam.org/) databases.

### Phylogenetic analysis

Phylogenetic analysis was performed using the MEGA 11.0 program [35]. The protein sequences were aligned using the ClustalW method in MEGA 11.0 with the following parameters: pairwise alignment gap opening penalty of 10, gap extension penalty of 0.1, multiple alignment gap opening penalty of 10, gap extension penalty of 0.2, the use negative matrix was turned off, and the delay divergence cutoff (percentage) was set at 30. The resulting alignment was then used to construct an unrooted tree using the neighbor-joining method with bootstrap analysis (*n* = 1000, amino acid, Poisson model, rate and patterns: uniform rates, data subset to use: pairwise deletion, traditional tree without modification for graphics). Statistical tests were performed, and the bootstrap values for the branches are shown in the generated tree.

### Gene expression analysis

We used QRT-PCR to examine the transcriptional levels of genes. In tea plants, the relative gene expression levels were determined using a 2^−ΔΔ Ct^ method. The house-keeping gene *CsGAPDH* of tea plants was employed as an internal standard to normalize cDNA concentrations. The protocols for this method has been described in detail in our previous study [19]. As *CsSERPIN1* gene is absent in wild-type Arabidopsis, we employed a standard curve method rather than 2^−ΔΔ Ct^ method to quantify the expression levels of *CsSERPIN1* in both wild-type and *CsSERPIN1*-heteroexpressed Arabidopsis plants. This method does not depend on the *CsSERPIN1* expression of wild-type plants as calibrator samples. Therefore, the relative expression of *CsSERPIN1* in the heteroexpressed lines can be accurately calculated even in the absence of *CsSERPIN1* in wild-type Arabidopsis. For a comprehensive understanding of the standard curve method, please refer to Wong and Medrano [36]. Specifically, we used serial dilutions of a specific cDNA standard to generate a standard curve, and then applied the cycle threshold (Ct) values of the samples to the curve to calculate the relative expression levels of *CsSERPIN1*. The *CsSERPIN1* relative expression level in Arabidopsis is the transcript level of *CsSERPIN1* relative to the Arabidopsis house-keeping gene glyceraldehyde-3-phosphate dehydrogenase (*AtGAPDH*). QRT-PCR primers for the house-keeping and tested genes, including *CsSERPIN1*, *CsSERPIN2* (LOC114256809), *CsSERPIN3* (LOC114271199), and *CsSERPIN4* (LOC114271172) are all listed in Table S4 (see online supplementary material).

### Plant treatments

For herbivory treatment, two third-instar tea geometrid larvae were allowed to feed on the second and third fully expanded leaves, which were consistent with the tissue used for the herbivore resistance evaluation of tea plants. The leaf and the larvae were covered with a small mesh pocket (9 cm × 11 cm) to prevent larvae from escaping. Leaves covered with empty bags were used as control. The whole leaves were harvested at specified time intervals after herbivore attack (*n* = 5–6).

For the treatment of defense signaling molecules, JA, ABA, SA, or 1-aminocyclopropane-1-carboxylic acid (ACC, for ethylene treatment) (Solarbio, Beijing, China) was first dissolved in ethanol of a small volume, and further diluted in the sterilized Milli-Q water to get working solutions with a concentration of 200 μM for JA, ABA, 1 mM for SA, or 100 μM for ACC. The prepared working solutions were then thoroughly sprayed onto the leaf surface. The control plants were treated similarly with Milli-Q water and ethanol indicated above. The second leaves were harvested at specified time intervals after treatments (*n* = 5).

For pathogen infection, the spore suspension of *Colletotrichum camelliae* was inoculated onto tea leaves, which were mechanically pieced as described preciously [37]. Sterile water was inoculated as a control. The infected leaves were harvested at specified time intervals after treatments (*n* = 5).

For cold and heat treatment, tea plants were treated at 4°C (cold stress) or 38°C (heat stress). The control plants were treated at 22°C. The apical second and third young leaves were harvested at specified time intervals after treatments (*n* = 5).

### CsSERPIN1 expression and purification in *E. coli*

The ORF of *CsSERPIN1* was inserted into a pMAL-c2X vector, in frame with a maltose binding protein (MBP) tag, and then transformed into *E. coli* strain BL21(DE3). After incubation in ampicillin-containing LB medium for 20 h at 37°C, the culture cells were diluted 1:50 and grown until OD600 was 0.7. IPTG was added to a final concentration of 1 mM, and the cells were further incubated at 16°C and 140 rpm overnight for the protein expression. The fused proteins were purified by MPB-binding resin (New England BioLabs, Ipswich, Massachusetts, USA) following the manufacturer instructions. Concentrations of purified protein were quantified using the Bradford method (Bradford Protein Assay Kit, Solarbio, Beijing, China). The purified proteins were further confirmed by immunoblot blot using an anti-MBP monoclonal antibody (Genscript, Nanjing, China) and a horseradish peroxidase-conjugated secondary antibody (Solarbio, Beijing, China).

### Larval gut extraction

To extract the larval gut, fifth-instar larvae were cold-anesthetized and dissected. The midguts and contents from ten larvae were pooled together, weighed in tubes, and subsequently homogenized in a volume of 0.15 M sodium chloride equivalent to the gut weight. Homogenates were centrifugated, and supernatants were collected. The total protein content in supernatants was determined using a Bradford assay (Bradford Protein Assay Kit, Solarbio, China).

### CsSERPIN1 inhibitory activity

The ability of CsSERPIN1 to inhibit the activities of chymotrypsin and trypsin was determined using previously described methods [27, 38, 39]. Briefly, the trypsin activity was analysed using α-*N*-benzoyl-_DL_-arginine-*p*-nitroanilide hydrochloride (BApNA) as a substrate with a concentration of 1.5 mM in 0.8% (v/v) DMSO. The enzyme (4.7 × 10^−5^ M) and purified CsSERPIN1 protein (3–18 μg) were thermo-equilibrated at 37°C for 10 min before addition of substrate to start the reaction. The buffer used was 50 mM Tris–HCl, pH 8.0. After 37°C incubating for 20 min, 30% acetic acid (v/v) was added to stop the reaction. The protease activities were measured by the absorbance at 410 nm. For gut protease activities, the larval gut protein extracts (10 μg) and the substrate were incubated in buffer for 20 min. Appropriate blanks were uses in all the assays. Chymotrypsin protease was quantified similarly as described above, except *N*-succinyl-alanine–alanine-proline-phenylalanine-*p*-nitroanilide (SAAPFpNA) was used as a substrate.

### Transient overexpression of *CsSERPIN1* in tea plants

The ORF of *CsSERPIN1* was cloned into the pBWA(V)HS-GFP vector and the resulting pBWA(V)HS-*CsSERPIN1*-GFP plasmid was used to transform *A. tumefaciens*. Third leaves of 3-year-old tea plants were infiltrated with the transformed *Agrobacterium* using the *A. tumefaciens*-mediated infiltration method [40]. The empty vector pBWA(V)HS-GFP and infiltration buffer were infiltrated as controls. The entire leaves were harvested for determination of *CsSERPIN1* overexpression using QRT-PCR (*n* = 5).

### Heterologous expression of *CsSERPIN1* in Arabidopsis

*CsSERPIN1* was cloned into a pBWA(V)HS vector carrying a FLAG epitope tag to yield an heteroexpression construct, pBWA(V)HS-*CsSERPIN1*-flag. The recombinant vector was then transferred into *A. thaliana* Columbia-0 via *Agrobacterium*-mediated transformation, as described previously [41]. Two T_2_ homozygous lines (L2 and L5) were selected for subsequent experiments. The expression levels of *CsSERPIN1* were confirmed by QRT-PCR and immunoblot assays. An anti-FLAG antibody (Sigma-Aldrich, Darmstadt, Germany) and a horseradish peroxidase-conjugated secondary antibody (Solarbio, Beijing, China) were used for the immunoblot assays.

### Effects of CsSERPIN1 on herbivore performance

#### Effect of purified CsSERPIN1 on tea geometrid

Freshly prepared artificial diet was cut into small pieces (10 × 10 × 10 mm) and separated into petri dishes [42]. Twenty microliters of 800 μg ml^−1^ purified CsSERPIN1-MBP proteins were added to each diet, resulting in a concentration of 16 μg ml^−1^ in the diet. The amount of CsSERPIN1-MBP protein added was based on its inhibitory effects, which was measured in *in vitro* assays. As shown in Fig. 3b, 12 to 18 μg of CsSERPIN1-MBP protein in 1 ml reaction buffer (12–18 μg ml^−1^) resulted in strong inhibition of trypsin and chymotrypsin activities, with more than 70% and 20% inhibition, respectively. Therefore, 16 μg of CsSERPIN1-MBP protein was introduced into 1 cm^3^ artificial diet (16 μg ml^−1^) to test its inhibitory effects on larva performance. Groups of ten three-day-old tea geometrid caterpillars of similar size were pre-starved for 6 h and introduced into the petri dishes. The diets with proteins were replaced every half-day to ensure constant protein activity and sufficient fresh food source for the caterpillars. Diets with MBP or distilled water were used as controls. Larval weight was quantified 5 d and 8 d after the start of the assay, and the pupal length and weight were measured after larval pupation (*n* = 24–30).

#### Effect of overexpressed CsSERPIN1 on tea geometrid

Two three-day old tea geometrid caterpillars of similar size were introduced to feed on the control, empty vector, or *CsSERPIN1-*trasnformed tea plants. The larvae were confined to the leaves of each plant with a small mesh pocket (9 cm × 11 cm) to prevent escape. The leaves were replaced once half of them were consumed. Larval weight was quantified 5 d after the start of the assay (*n* = 26).

#### Effect of heteroexpressed CsSERPIN1 on fall armyworm

A second-instar fall armyworm larva was pre-weighted and then allowed to feed on the wild type and two *CsSERPIN1*-heteroexpressed lines. Each plant and larva were individually confined in a transparent plastic cup with a lid (10 cm in height and 8 cm diameter) to prevent larvae from escaping. The plants were replaced once half of leaves were consumed. Larval weight was quantified 5 and 7 d after the start of the assay (*n* = 25).

### Data analysis

Differences in herbivore growth, gene expression and protease activities in different plant lines, treatments, or time points were determined by one-way/two-way ANOVA. Multiple comparisons were further conducted by False Discovery Rate-corrected Least Squares Means [43]. The ‘cor. Test’ function in R was used to analyse the correlations between expression levels of *CsSERPIN1/2/3/4* and herbivore growth [44]. All analyses were conducted using R 4.1.0 software.

## Acknowledgements

We thank Prof. Liang Chen for insightful input and valuable scientific suggestions, Prof. Dr Xinchao Wang, Lu Wang and Yuchun Wang for kindly supplying experimental materials, Xiwang Li and Jianying Jin for looking after the insects and plants. This research was supported by National Natural Science Foundation of China (31272053, 31901898), Central Public-interest Scientific Institution Basal Research Fund (Y2023PT03, 1610212019001), and the Elite Youth Program of Chinese Academy of Agricultural Sciences for Meng Ye.

## Author contributions

M.Y. and X.S. designed the research; M.Y., C.L., N.L., C.Y., M.L., Z.X. and S.L. performed experiments; M.Y. and X.S. analysed data and wrote the paper. All authors approved the article.

## Data availability

All the relevant data are provided in this article and its supplementary information. The original readcount and fpkm of RNA sequencing data for CsSERPINs and CsLOXs reported in this paper is deposited in Data S1. All the materials supporting the findings of this study are available from the corresponding authors upon request.

## Conflict of interests

None declared.

## Supplementary data

Supplementary data is available at *Horticulture Research* online.

## Supplementary Material

Web_Material_uhad178
