# Supplementary material for: A constitutive serine protease inhibitor suppresses herbivore performance in tea (Camellia sinensis)
Source: Hortic Res. 2023 Sep 1;10(10):uhad178. doi: 10.1093/hr/uhad178 (PMC10585712; doi:10.1093/hr/uhad178)
Supplement: Web_Material_uhad178 [file web_material_uhad178.zip › HR_supplementary information.docx]

**Supplementary Information for**

**A constitutive serine protease inhibitor suppresses herbivore performance in tea (*Camellia sinensis*)**

Meng Ye*, Chuande Liu, Nana Li, Chenhong Yuan, Miaomiao Liu, Zhaojun Xin, Shu Lei, Xiaoling Sun*

Key Laboratory of Biology, Genetics and Breeding of Special Economic Animals and Plants, Ministry of Agriculture and Rural Affairs, National Center for Tea Plant Improvement, Tea Research Institute, Chinese Academy of Agricultural Sciences, Hangzhou, China

*Correspondence: Meng Ye ([mengye@tricaas.com](mailto:mengye@tricaas.com)); Xiaoling Sun ([xlsun@mail.tricaas.com](mailto:xlsun@mail.tricaas.com))


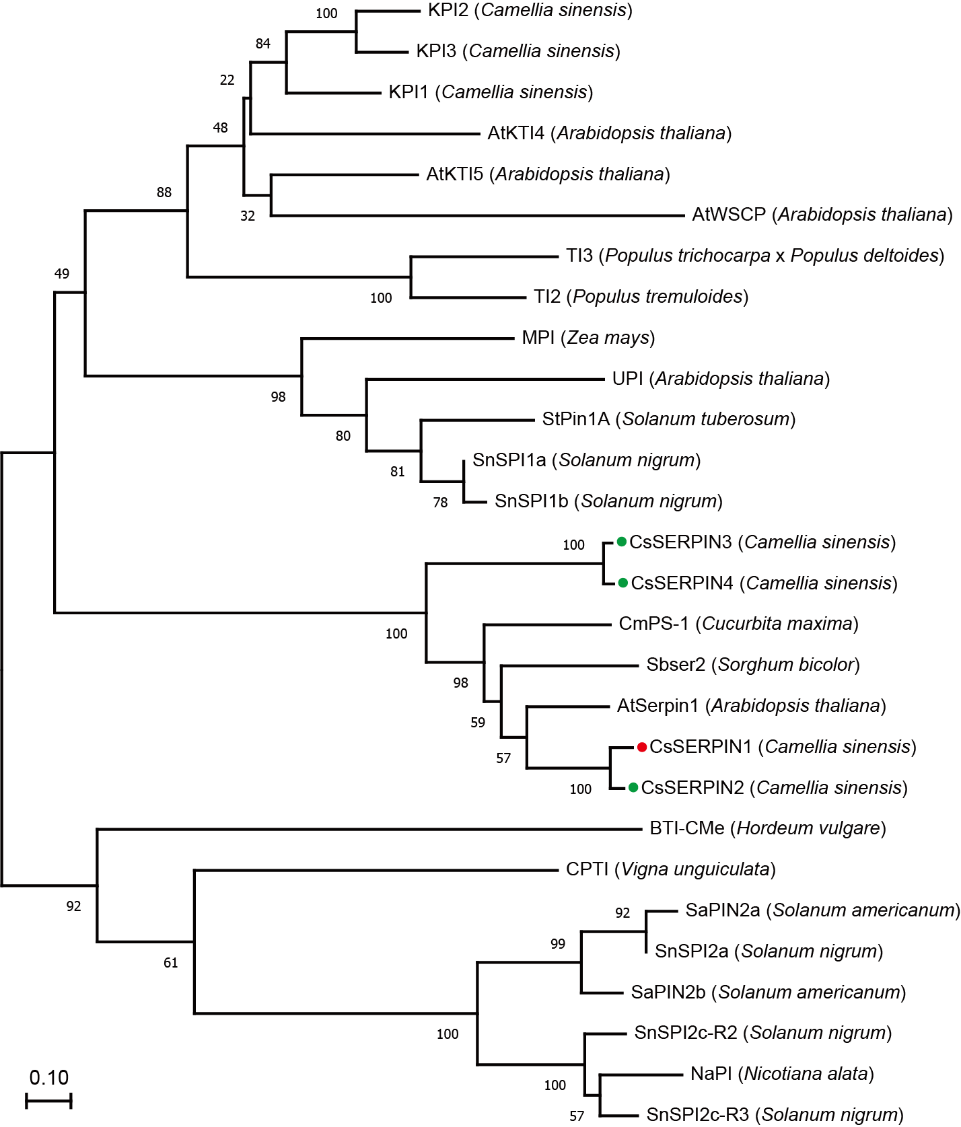


**Figure S1 Phylogenetic analysis of herbivore-related SERPINs from various** **plant species.** An unrooted tree was constructed using the neighbor-joining method based on the alignment of protein sequences. Confirmation of the tree topology was achieved through bootstrap analysis with 1,000 replicates using the MEGA software (default settings, except for the bootstrap value replicates). The bootstrap values for the branches are provided, and the scale bar represents 0.1 amino acid substitutions per site in the primary structure. Accession numbers for the sequence data in the phylogenetic tree can be found in the GenBank data libraries: AtSerpin1 (NP_001320925); AtKTI4 (NP_565061); AtKTI5 (NP_173228); AtWSCP (NP_177373); UPI (NP_199171); TI2 (AAK32690); TI3 (AAQ84216); KPI1 (MK057519); KPI2 (MK057520); KPI3 (MK057521); CmPS-1 (AAG02411); Sbser2 (XP_002466823); BTI-CMe (XP_044974724); MPI (NP_001105449); CPTI (AAO43979); SaPIN2a (AAS82779); NaPI (AAF14181); StPin1A (ACZ04396); SnSPI1a (ADP05502); SnSPI1b (ADP05503); SnSPI2a (ADP05505); SnSPI2c-R2 (ADP05506); SnSPI2c-R3 (ADP05507); SaPIN2b (AAL54921).


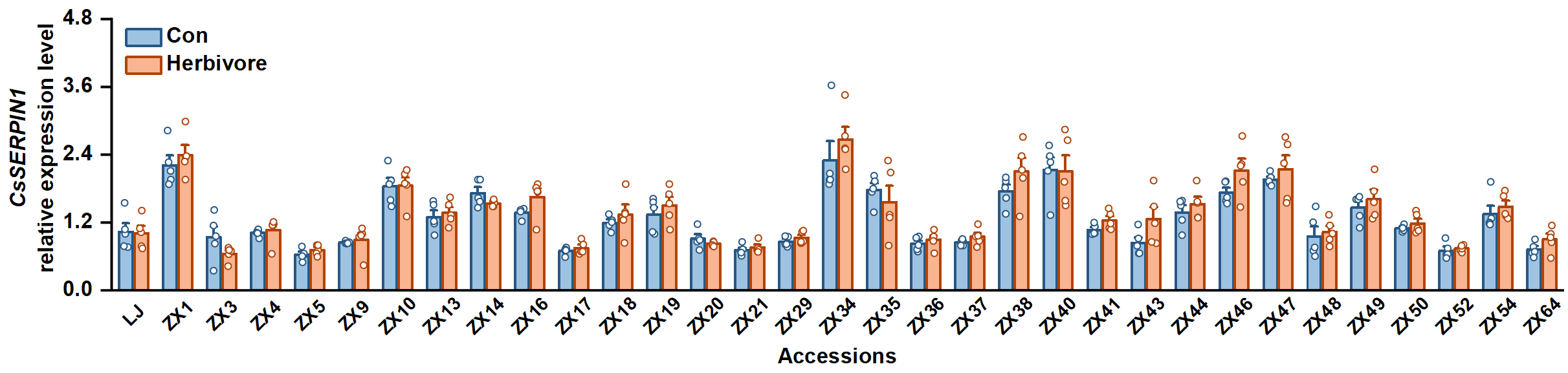


**Figure S2 Expression levels of *CsSERPIN1* in different tea accessions under herbivore attack.** Transcript levels of *CsSERPIN1* (+ SE, *n* = 5) in 33 tea accessions after 24 h herbivory.

**Table** **S1 Analysis of variance (ANOVA) of herbivore performance on different tea accessions.**

Overall ANOVA:

|  | DF | Sum of Squares | Mean Square | F Value | Prob>F |
| --- | --- | --- | --- | --- | --- |
| Model | 32 | 333366.1 | 10417.69 | 16.62287 | 8.15E-79 |
| Error | 1725 | 1081072 | 626.7084 |  |  |
| Total | 1757 | 1414438 |  |  |  |

Multiple comparison through FDR-corrected Least Squares Means:

| Accessions | Mean | Groups | | | | | | | | | | | |
| --- | --- | --- | --- | --- | --- | --- | --- | --- | --- | --- | --- | --- | --- |
| ZX16 | 94.23654 | A |  |  |  |  |  |  |  |  |  |  |  |
| ZX13 | 90.70962 | A | B |  |  |  |  |  |  |  |  |  |  |
| ZX18 | 88.63962 | A | B | C |  |  |  |  |  |  |  |  |  |
| ZX54 | 84.06792 | A | B | C | D |  |  |  |  |  |  |  |  |
| ZX34 | 79.06275 | A | B | C | D | E |  |  |  |  |  |  |  |
| ZX41 | 78.66667 | A | B | C | D | E |  |  |  |  |  |  |  |
| ZX47 | 77.26863 | A | B | C | D | E |  |  |  |  |  |  |  |
| ZX21 | 77.078 | A | B | C | D | E |  |  |  |  |  |  |  |
| ZX20 | 75.59818 |  | B | C | D | E | F |  |  |  |  |  |  |
| ZX44 | 75.3549 |  | B | C | D | E | F | G |  |  |  |  |  |
| ZX38 | 74.466 |  | B | C | D | E | F | G | H |  |  |  |  |
| ZX49 | 74.2037 |  | B | C | D | E | F | G | H |  |  |  |  |
| ZX35 | 73.77358 |  | B | C | D | E | F | G | H |  |  |  |  |
| LJ | 73.41569 |  | B | C | D | E | F | G | H |  |  |  |  |
| ZX17 | 72.72364 |  | B | C | D | E | F | G | H |  |  |  |  |
| ZX43 | 70.46226 |  |  | C | D | E | F | G | H | I |  |  |  |
| ZX52 | 68.44 |  |  |  | D | E | F | G | H | I |  |  |  |
| ZX46 | 65.21852 |  |  |  |  | E | F | G | H | I | J |  |  |
| ZX50 | 62.91273 |  |  |  |  | E | F | G | H | I | J | K |  |
| ZX29 | 60.72909 |  |  |  |  | E | F | G | H | I | J | K |  |
| ZX9 | 58.10962 |  |  |  |  |  | F | G | H | I | J | K |  |
| ZX36 | 58.02778 |  |  |  |  |  | F | G | H | I | J | K |  |
| ZX37 | 57.96 |  |  |  |  |  | F | G | H | I | J | K |  |
| ZX3 | 57.71455 |  |  |  |  |  | F | G | H | I | J | K |  |
| ZX14 | 56.83962 |  |  |  |  |  |  | G | H | I | J | K | L |
| ZX4 | 55.93269 |  |  |  |  |  |  |  | H | I | J | K | L |
| ZX5 | 53.90926 |  |  |  |  |  |  |  |  | I | J | K | L |
| ZX64 | 52.60189 |  |  |  |  |  |  |  |  | I | J | K | L |
| ZX40 | 49.75455 |  |  |  |  |  |  |  |  |  | J | K | L |
| ZX1 | 47.93519 |  |  |  |  |  |  |  |  |  | J | K | L |
| ZX19 | 46.79091 |  |  |  |  |  |  |  |  |  |  | K | L |
| ZX10 | 45.30364 |  |  |  |  |  |  |  |  |  |  | K | L |
| ZX48 | 38.74259 |  |  |  |  |  |  |  |  |  |  |  | L |

**Table S2 ANOVA of *CsSERPIN1* expression levels on different tea accessions.**

Overall ANOVA:

|  | DF | Sum of Squares | Mean Square | F Value | Prob>F |
| --- | --- | --- | --- | --- | --- |
| Model | 32 | 19.98281 | 0.624463 | 4.323287 | 2.71E-10 |
| Error | 163 | 23.544 | 0.144442 |  |  |
| Total | 195 | 43.52681 |  |  |  |

Multiple comparisons through FDR-corrected Least Squares Means:

|  | Mean | Groups | Groups | Groups | Groups | Groups |
| --- | --- | --- | --- | --- | --- | --- |
| ZX14 | 2.005549 | A |  |  |  |  |
| ZX48 | 1.736667 | A | B |  |  |  |
| ZX10 | 1.65706 | A | B | C |  |  |
| ZX40 | 1.601146 | A | B | C | D |  |
| ZX1 | 1.589165 | A | B | C | D |  |
| ZX19 | 1.528111 | A | B | C | D | E |
| ZX4 | 1.500725 | A | B | C | D | E |
| ZX47 | 1.39302 | A | B | C | D | E |
| ZX64 | 1.383847 | A | B | C | D | E |
| ZX34 | 1.304939 | A | B | C | D | E |
| ZX49 | 1.296792 | A | B | C | D | E |
| ZX46 | 1.276428 | A | B | C | D | E |
| ZX38 | 1.232699 | A | B | C | D | E |
| ZX13 | 1.145047 |  | B | C | D | E |
| ZX50 | 1.128511 |  | B | C | D | E |
| ZX36 | 1.115732 |  | B | C | D | E |
| LJ | 1.079226 |  | B | C | D | E |
| ZX9 | 1.057489 |  | B | C | D | E |
| ZX16 | 1.047951 |  | B | C | D | E |
| ZX54 | 0.999404 |  | B | C | D | E |
| ZX37 | 0.979259 |  | B | C | D | E |
| ZX43 | 0.954559 |  | B | C | D | E |
| ZX44 | 0.938862 |  | B | C | D | E |
| ZX3 | 0.923949 |  | B | C | D | E |
| ZX41 | 0.905963 |  | B | C | D | E |
| ZX35 | 0.866185 |  |  | C | D | E |
| ZX18 | 0.84167 |  |  | C | D | E |
| ZX52 | 0.836063 |  |  | C | D | E |
| ZX5 | 0.834786 |  |  | C | D | E |
| ZX17 | 0.821212 |  |  | C | D | E |
| ZX29 | 0.809362 |  |  | C | D | E |
| ZX21 | 0.773749 |  |  |  | D | E |
| ZX20 | 0.71853 |  |  |  |  | E |

**Table S3 Detailed information of the tea accessions used in this study.**

| Accessions | Origin | Registered name |
| --- | --- | --- |
| ZX10 | Local population ‘Qimen’ | Zhongcha 105 |
| ZX19 | Progeny of local population ‘Meizhou Qunti’ in Guangdong province | NA |
| ZX48 | Progeny of local population ‘Yongzhou Qunti’ in Hunan province | NA |
| ZX13 | Local population ‘Middle/small-leaved Qunti’ in Sichuan province | Zhongcha 308 |
| ZX16 | Local population ‘Qimen Chuye’ | Zhongcha 313 |
| ZX18 | Local population ‘Mangshan’ | Zhongcha 307 |

**Table S4 QRT-PCR primers of target genes.**

| Name | Forward primer sequence (5′–3′) | Reverse primer sequence (5′–3′) |
| --- | --- | --- |
| *CsSERPIN1* | TCACCACCTCCCATACCAAG | CTTGACCGGAGAAAGGCAAG |
| *CsSERPIN2* | ACCAAGAAGTAGAAGTGGGAG | CATCTCGGTAAGCCCTCCG |
| *CsSERPIN3* | AAAGGGATTGGGATTGGTCT | AAAGCCCTCTGCTGTATCAC |
| *CsSERPIN4* | CAAGAGGTTGGAAAGAGCAT | CTAGCAGCCACCATGTTTAG |
| *CsLOX7* | AAGCAAGACTCAATCACACCAT | GGCGTTCACCAACTCGTTA |
| *CsJAZ2* | TCGTCGAGCTTCTCTCAGAC | CGTCACCGGAGTTCCATTTC |
| *CsUGT87E7* | GGGCACATCAACCCCATGAT | GGGCAAGACGTTTGGGATTG |
| *CsACS1* | GAATAATCCCAAAGCCTCCA | AGCCACACCCTTTCTGAACT |
| *CsSnRK2.1* | CAGCTCAAGCCATGTATTACAGGAG | CATCTTCTTCTTCCTCGGCTTCTTCG |
| *CsCBF1* | AGAAATCGGATGGCTTGTGT | TTGTCGTCTCAGTCGCAGTT |
| *CsHSP90* | TCGAGGAGGATGAGGAAGCA | TCCACAACACACGCCCATTA |
| *CsGSTU19* | TTTGGAGGAGACAGTGTTGG | GGTGGCAGATGTTCTTTGATG |
| *CsGAPDH* | ATACCACGTCATCCTCGGT | ACTTATGATGAAATCAAAGCTGC |
| *AtGAPDH* | AGGTCAAGCATTTTCGATGC | AACGATAAGGTCAACGACACG |
